# Predictors of Epicardial Fat Volume Decrease after Dapagliflozin Treatment in Patients with Type 2 Diabetes

**DOI:** 10.3390/medicina58010021

**Published:** 2021-12-23

**Authors:** Adina Braha, Alin Albai, Bogdan Timar, Daniela Cipu, Lucian Vasiluță, Ovidiu Potre, Romulus Timar

**Affiliations:** 1 Second Department of Internal Medicine, “Victor Babeș” University of Medicine and Pharmacy, 300041 Timisoara, Romania; braha.adina@umft.ro (A.B.); bogdan.timar@umft.ro (B.T.); l.vasiluta@yahoo.com (L.V.); timar.romulus@umft.ro (R.T.); 2 Department of Orthopedics-Traumatology, Urology and Medical Imaging, “Victor Babeș” University of Medicine and Pharmacy, 300041 Timisoara, Romania; danscipu@yahoo.com; 3 First Department of Internal Medicine, “Victor Babeș” University of Medicine and Pharmacy, 300041 Timisoara, Romania; potre.ovidiu@umft.ro

**Keywords:** epicardial fat volume predictors, dapagliflozin, product of triglycerides and glucose (TyG)

## Abstract

*Background and Objectives*: Dapagliflozin treatment proved to reduce the epicardial fat volume (EFV) in patients with type 2 diabetes (T2D). Despite the reduction in EFV being associated with improved diastolic function in patients with T2D, EVF is not routinely evaluated in T2D because it is costly and involves radiation exposure. This study aims to identify biomarkers that predict EFV reduction after dapagliflozin treatment in patients with T2D. *Materials and Methods*: In a prospective, observational, consecutive-case enrollment scenario, 52 patients with T2D were initiated on dapagliflozin 10 mg q.d. as part of the standard of care. At enrollment and after six months of dapagliflozin treatment, patients were evaluated using cardiac ultrasonography, native computer tomography, transient liver elastography, and metabolic lab tests. In addition, the atherogenic index of plasma (AIP), atherogenic coefficient (AC), triglyceride glucose index (TyG), cardiac risk ratio (CRR), and visceral abdominal index (VAI) were calculated. *Results*: Higher AIP (*r* = 0.28; *p* = 0.04), CRR (*r* = 0.28; *p* = 0.04), and TyG (*r* = 0.32; *p* = 0.01) are associated with more important reductions in the EFV. A lower conicity index (β = −0.29; *p* = 0.03), visceral fat volume at the 4^th^ vertebrae (L4VFV) (β = −0.32; *p* = 0.02), left atrium volume (β = −3.08; *p* = 0.003), and right ventricle diameter (β = −2.13; *p* = 0.04) are associated with higher reductions in the EFV after six months of dapagliflozin treatment. A valid performance for predicting clinically relevant decreases in EFV after dapagliflozin treatment was observed for AIP (AUROC = 0.903; Youden = 0.732; *p* < 0.001), CRR (AUROC = 0.772; Youden = 0.595; *p* = 0.004), TyG (AUROC = 0.957; Youden = 0.904; *p* < 0.001), and VAI (AUROC = 0.898; Youden = 0.712; *p* < 0.001). *Conclusion*: Higher initial EFV values are associated with more important reductions in EFV in patients with T2D treated for six months with dapagliflozin. TyG values have the best prediction performances for EFV reduction, having the highest sum of sensitivity and specificity at the 0.904 threshold level. AIP, CRR, VAI, conicity index, L4VF, left atrium volume, and right ventricle volume are valid biomarkers for a decrease in EFV after dapagliflozin treatment in diabetes patients.

## 1. Introduction

Worldwide, more than 463 million adults live with diabetes, of which, approximately 90% have type 2 diabetes (T2D) [1]. Among people with diabetes, the leading cause of mortality is represented by cardiovascular diseases (CVD) [2]. Irrespective of other cardiovascular risk factors, such as hypertension, dyslipidemia, age, or coronary artery disease, diabetes increases the risk of developing heart failure from two- to five-fold (in men and women, respectively) [3,4]. In addition, obesity is an important cardiovascular risk factor frequently associated with T2D, thus increasing the probability of developing heart failure by mechanisms of altered cardiac metabolism and neurohumoral and adipokine secretion [5,6,7,8]. However, recent studies have proved that adipose tissue depots have different atherogenic potentials. The first incriminated in atherosclerotic CVD is the epicardial and pericardial fat tissue surrounding the coronary arteries, accelerating cardiac atherosclerosis and heart dysfunction [7,9]. This phenomenon of cardiac atherosclerosis augmentation was observed mainly in T2D patients [10,11,12] who have an increased level of epicardial and pericardial fat [13].

Moreover, epicardial adipose tissue in T2D patients is associated not only with atherosclerosis [14] but also with diastolic dysfunction [15] and cardiovascular events [16], thus making epicardial adipose tissue an emergent hypothetical therapeutic target. Regarding the management of T2D, according to the latest guidelines [17], sodium-glucose-2 co-transporter inhibitors (SGLT2i) should be the first pharmaceutical step after metformin in patients with T2D and heart failure. In a previous study that supports the recommendations of the American Diabetes Association (ADA) guidelines, dapagliflozin, an SGLT2i, demonstrated beneficial effects in type 1 diastolic dysfunction remission, especially in T2D patients with a diabetes evolution of up to 8 years [18] in association with an epicardial adipose fat volume (EFV) decrease, beyond the glycemic control [19].

Despite the evidence that the epicardial fat depot has a significant role in CVD, its assessment remains difficult in clinical practice. Therefore, the study’s main aim is to evaluate which biomarkers are associated with decreases in EFV reduction after dapagliflozin treatment in patients with T2D. Identifying these biomarkers will allow for better therapeutic results by defining the patient characteristics, which will have additional benefits after dapagliflozin treatment.

## 2. Materials and Methods

### 2.1. Study Design and Patients

In a prospective, observational, consecutive-case enrollment scenario, 52 patients with T2D were initiated on dapagliflozin 10 mg q.d. as part of the standard of care.

The initial database review pointed out 80 patients with T2D, eligible for initiation of an SGLT2i (dapagliflozin), by their diabetologist from the “Pius Brînzeu” Diabetes Clinic Center, according to the standard of care. The study inclusion criteria were: age > 18 years, HbA1c > 7%, estimated glomerular filtration rate (eGFR) > 60 mL/min/1.73 m^2^, no history of chronic hepatitis, alcohol abuse, or significant cardiovascular disease. The exclusion criteria were: above criteria not met, normal weight, ongoing or planned pregnancy, heart failure with left ventricular ejection fraction below 40% or another significant heart disease, history of diabetic ketoacidosis, or urinary infections, which were not eligible for dapagliflozin treatment. In addition, the refusal of participation in this study, discontinuing the therapy with dapagliflozin, or missing the appointments of computer tomography excluded some patients from the trial. Sixty-eight patients were eligible for inclusion in the study and started treatment with dapagliflozin, but 15 patients did not reach the end of the follow-up period in the study due to discontinuation of treatment, missing the follow-up medical visits, and adverse effects related to dapagliflozin (urinary infections, euglycemic ketoacidosis). One missed the second appointment of computer tomography. Finally, 52 patients finalized all of the investigations in the study, and 28 patients were excluded before or during the study.

### 2.2. Ethics Statement

Prior enrollment, the study protocol, and the patient’s informed consent obtained the approval (no. 28/29.03.2018) of the Ethics Committee of “Pius Brînzeu” Emergency Hospital. The study was conducted according to the Helsinki Declaration. Prior to the first study-related procedure, all of the enrolled patients provided written informed consent.

### 2.3. Methods

Every three months, patients were scheduled for medical follow-ups at the Diabetes Center, according to the standard of care. The study-related patient evaluations were conducted before starting the therapy with dapagliflozin and at a six-month follow-up visit. These evaluations included biological routine tests and imagistic evaluations, cardiac ultrasonography, native computer tomography (CT) to assess subcutaneous and visceral adipose tissue volumes at different levels (epicardial, abdominal at the 4th vertebrae), and liver vibration controlled transient elastography (Fibroscan Echosens) to assess the degree of fibrosis (fibroscan) and hepatic steatosis (CAP) in a fasting state. In addition, age, medical history, diabetes duration, hypertension, outpatient treatment, and diabetes complications were recorded from anamnesis or medical files. Furthermore, weight, height, and waist circumference (waist) were measured at each follow-up visit.

A native CT scan was performed to evaluate patients’ adipose tissue by using the available equipment, Philips MX 16-Slice CT Scanner System, Philips Medical Systems (Cleveland) Inc. The scan was performed axially, from cranially to caudally, from the thoracic opening to the pelvis level, with multiple sections of 1 mm, at high resolution, with the following exposure parameters: 120 kV and 40 mA. Afterward, the images were processed using a digital analysis software 3D Slicer 4.8.1 r26813 [20], which allows for the anatomical selection of the tissues and their differentiation according to density; for adipose tissue was used a threshold between −30 and −190 Hounsfield Units [21]. Finally, epicardial fat volume (EFV) and visceral fat volume at the fourth vertebrae (L4VFV) were analyzed after correction for body surface area.

Used Definitions

Body mass index (BMI) = weight/height^2^

Body surface area = Weight^0.425^ × Height^0.725^ × 0.007184 [22]

Conicity index = 0.109^−1^ × waist (meters) × (weight [kg]/height [meters]) − 1/2 [23]

Atherogenic index of plasma (AIP) = log [TG/HDLc] [24]

Cardiac risk ratio (CRR) = TC/HDLc [25]

Atherogenic coefficient (AC) = non-HDLc/HDLc [24]

Product of triglycerides and glucose (TyG) = ln [Fasting triglyceride (mg/dL) × Fasting glucose (mg/dL)]/2 [26]

Visceral adiposity index (VAI) [27]:

Females VAI = (waist/(36.58 + (1.89 × BMI))) × (TG/0.81) × (1.52/HDLc),

Males VAI = (waist/(39.68 + (1.88 × BMI))) × (TG/1.03) × (1.31/HDLc),

where waist is expressed in cm, BMI in kg/m^2^, TG and HDLc in mmol/L.

In recent studies, the cut-offs AIP > 0.24, CRR > 5, and AC > 3 were considered as cardiovascular risk criteria [25,28].

### 2.4. Statistical Analysis

GNU PSPP (Version 1.4.1, Software Foundation. Boston, MA, USA) and MedCalc^®^ Statistical Software version 20.014 (MedCalc Software Ltd., Ostend, Belgium) were used to perform the statistical analysis. After testing for normal distribution, we calculated the mean ± standard deviation for continuous numerical variables with Gaussian distribution, and the median [interquartile range] for non-parametric numerical variables. To evaluate the significances of the differences between the first and last medical follow-up, the t-test (variables with Gaussian distribution) and Mann–Whitney U-test (variables with non-parametric distributions) were performed, respectively.

The strength of association between each atherogenic index (AIP, CRR, TyG, VAI) and studied variable was assessed using the Pearson correlation coefficient for variables with Gaussian distribution and the Spearman rank correlation for non-parametric variables. Afterward, linear univariate regression analysis was performed to assess the influence of patients’ baseline characteristics on EFV percentage decrease after six months of dapagliflozin. The standardized beta coefficient interpreted the degree of variation in the outcome variable for every 1 unit of change in the predictor variable. The strength of the relationship between the positively associated factors with the percentage decrease in EFV was evaluated using univariate and multivariate logistic regression models, where the EFV decrease was a dichotomous outcome (dependent variable). The variables retained in the regression model were evaluated for the association power with EFV decrease by receiver-operating characteristics (ROC) analysis through sensitivity, specificity, and Youden index. In addition, we compared the area under the model’s ROC curve with the non-discriminant one to analyze the predictive capacity’s statistical significance (the area under the ROC curve = 0.5).

In this study, a *p*-value of 0.05 was considered as the threshold for statistical significance.

## 3. Results

Out of the 52 patients, 32 (61.53%) were men. The mean age was 57.50 ± 10.35 years, the mean BMI was 34.55 ± 4.79 kg/m^2^, and the median diabetes duration of the patients was 7 years [0; 24]. Dapagliflozin (10 mg per day) represented an add-on therapy to baseline antidiabetic molecules in different combinations: metformin (94.23%), sulfonylureas (23.07%), basal insulin (17.30%), dipeptidyl-peptidase 4 inhibitors (DPP4i) (13.46%), and no glucagon-like peptide-1 agonists (GLP1a). In addition, 75% of patients had a statin, and 19.23% had fenofibrate included in their treatment.

In our study, 28/50 (56%) patients had AIP > 0.24, 24/51 (47%) CRR > 5, and 34/51 (66.6%) AC > 3.

After six months, dapagliflozin treatment was associated with significant decreases in body weight, BMI, waist circumference in men, glycemia, HbA1c, EFV, AIP, CRR, AC, TyG index, and CAP in parallel with significant increases in HDLc in men and the conicity index. The comparison of studied parameters at the six-month follow-up vs. baseline is presented in Table 1.

The AIP was directly and significantly associated with TC, glycemia, and HbA1c. CRR had a positive correlation with waist circumference, TC, TG, LDLc, and HbA1c, whereas TyG correlated with glycemia, HbA1c, TC. AIP, CRR, and VAI in both women and men who were negatively associated with HDLc. No significant correlation between the baseline EFV and studied indexes (AIP, CRR, TyG, or VAI) was found. Instead, the percentage by which EFV decreased was correlated with AIP (*r* = 0.28, *p* = 0.04), CRR (*r* = 0.28, *p* = 0.04), and TyG (*r* = 0.32, *p* = 0.01). The bivariate correlations between the studied parameters are presented in Table 2.

In the univariate linear regression model, the percentage decrease in EFV was not influenced by the patient’s age or diabetes duration, nor by the variation of weight, waist, BMI, lipids, uric acid, glycemia, HbA1c, liver steatosis, liver fibrosis, or VAI. Instead, the increase in CRR, AIP, and TyG with a standard deviation is expected to enhance the percentage decrease in EFV by 0.28 (*p* = 0.04), 0.29 (*p* = 0.04), and 0.33 (*p* = 0.02), respectively. In addition, with the increase in baseline EFV with a standard deviation, we expect a 0.73 increase (*p* < 0.001) in the EFV percentage reduction (Table 3).

The negative association of the conicity index, baseline value of L4VFV, percentage decrease in L4VFV, left atrium volume, and right ventricle diameter demonstrated that the higher these values are, the less important changes in the EFV percentage decrease are expected (Table 3).

**Figure 1:**
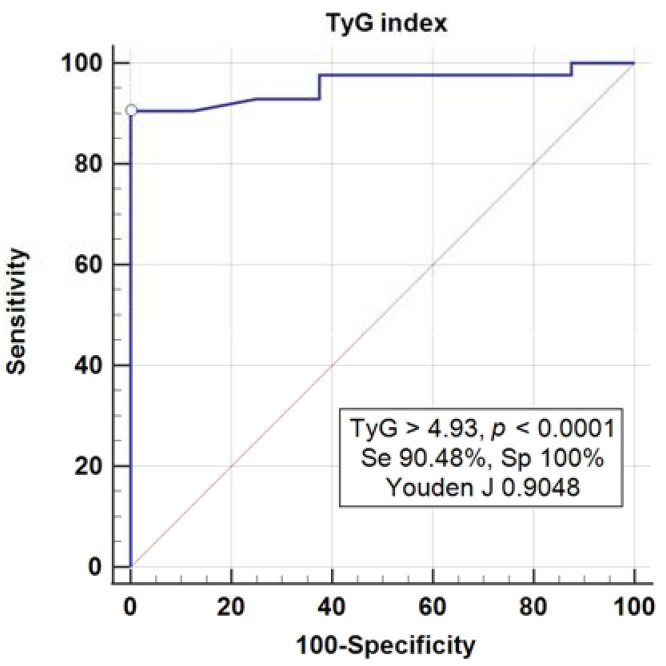
Receiver-operating characteristics (ROC) analysis for the baseline TyG index and the decrease in EFV (dichotomous event).

In the univariate logistic regression model and ROC curve analysis, the baseline value of EFV was strongly associated with the dichotomic event of the EFV decrease (AUC = 0.929, *p* < 0.0001, Youden index J = 0.7614, associated criterion > 22.3 cm^3^, sensitivity = 88.6%, specificity = 87.5%), suggesting that patients with initial values of EFV > 22.3 cm^3^ are expected to have a favorable outcome after dapagliflozin therapy. In addition, AIP, CRR, TyG, and VAI biomarkers were significantly associated with the dichotomic outcome, as presented in Table 4. However, after testing all of these biomarkers in a logistic multivariate regression model, TyG was the only biomarker retained in the model with the highest sensitivity and specificity for values > 4.93 (OR 29.5, 95% CI 10.2–85.2, Table 4, Figure 1).

## 4. Discussion

The present research is a prospective, consecutive-case, population-based study that investigated for the first time the predictive power of atherogenic biomarkers for the response to dapagliflozin therapy in a population of T2D patients. A previous study conducted by our team proved that dapagliflozin reduces the epicardial fat volume after only six months of treatment, regardless of glycemic control or bodyweight reduction [19].

More and more evidence shows that SGLT2i reduces the epicardial fat and other visceral fat depots [29,30], despite a moderate effect on bodyweight or HbA1c. In the EMPACEF study by Gaborit et al. 2021, 56 patients with T2D with normal left ventricle function showed a favorable response in bodyweight reduction and improved glycemic control after 12 weeks of empagliflozin, but no effect on myocardial or epicardial fat depots [29], probably due to the short time of therapy. In the LIGHT study, 36 patients showed changes in the total fat mass and visceral fat area, without an association with the effect on HbA1c levels, abdominal waist, or BMI after 52 weeks of luseogliflozin [30]. Luseogliflozin was efficient in reducing epicardial adipose tissue in a study by Bouchi R et al., with a 5.13% reduction after only 12 weeks of treatment [31]. In another small study, Yagi et al. demonstrated the reduction in epicardial fat thickness by 20.34% in 13 patients with T2D after 6 months of 100 mg canagliflozin daily [32]. Similarly, ipragliflozin reduced the epicardial fat depot by 12.75% in nine non-obese patients with T2D and high abdominal adiposity [33].

However, the exact mechanisms underlying the beneficial effects of dapagliflozin on epicardial fat depots are not fully understood. Epicardial adipose fat is associated with insulin resistance, high levels of pro-inflammatory cytokines, and a low differentiation ability of cells surrounding the heart. Rodriguez et al. obtained epicardial and subcutaneous fat biopsies from 52 patients treated with dapagliflozin who underwent heart surgery and showed an increased differentiation of stromal cells and reduced secretion of cytokines. The treatment of epicardial fat depots with dapagliflozin demonstrated an increased glucose uptake of differentiated adipocytes and a preserved insulin response, thus protecting the adjacent tissues [34].

The BMI is a simple and convenient index to assess the prevalence of obesity in the general population. However, a disadvantage of this index would be the inability to differentiate the visceral adipose tissue of subcutaneous adiposity. Studies have proved that people with similar BMI values can suffer from different comorbidities or have a variable cardiovascular risk [35]. Furthermore, over the past thirty years, several studies using CT or MRI have concluded that visceral adiposity more accurately reflects the cardiovascular risk than the subcutaneous adipose tissue [36]. Epicardial adipose tissue is also associated with atherosclerotic cardiovascular disease [14]. On the same note, visceral abdominal obesity is not always accurately reflected by measuring the abdominal waist. Emerging data suggest that combined high values of triglycerides with an increased waist, also called the hypertriglyceridemic waist, is more likely to be associated with visceral abdominal fat [37,38].

Our 6-month study analyzed the EFV response to SGLT2i and the association with several biomarkers (conicity index, AIP, AC, TyG index, CRR, and VAI) in a group of patients with T2D. These biomarkers are useful in practice to appreciate the effect of dapagliflozin on EFV without exposing patients to radiation like other investigations (CT scans, magnetic nuclear resonance).

The conicity index points out the distribution of body fat tissue; individuals with a double-cone body shape accumulate fat in the abdominal area, whereas those with a cylinder body shape have less adipose tissue around the central region [23]. The present study demonstrated a reverse association between the percentage decrease in EFV and the initial abdominal adiposity reflected by the conicity index.

VAI is a new index developed to evaluate both adipose tissue distribution and function [27]. In our study, VAI was not significantly changed after six months of dapagliflozin, whereas the conicity index showed a significant upward trend (*p* = 0.03). The conicity index indicates that the abdominal adiposity not only does not improve, it even amplifies, even if the patients lose weight. Instead, all of the atherogenic biomarkers studied (AIP, CRR, AC, TyG index) were significantly reduced after treatment. Therefore, we consider VAI to be a weak biomarker for evaluating a positive response of EFV to therapy.

Even though CAP was reduced significantly after 6 months of treatment, there was no association with EFV dynamics. Therefore, CAP is not a useful marker to predict both the improvement of liver steatosis and the reduction in the epicardial fat depot.

Recent evidence has framed AIP as a reliable biomarker for predicting the risk of coronary artery disease beyond individual lipid profiles in patients with T2D [39]. However, the mean AIP in our analysis was lower than in other studies, since the included patients did not have significant cardiovascular disease. For this reason, we assume that a baseline AIP > 0.02 is a valuable biomarker for predicting the EFV decrease after six months of SGLT2i (specificity 87.50%, sensitivity 85.71%) in T2D patients without coronary artery disease.

Both AC and CRR had the same power of association with our outcome, which is why we presented only the CRR predictive value. A CRR value > 4.03 indicates a favorable response for EFV decrease after treatment, with a sensitivity of 72.09% and the same specificity as AIP and VAI.

No association between the epicardial fat depot and age, diabetes duration, weight, waist, BMI, glycemia, HbA1c, uric acid, and the studied lipids were found. The most significant factor by far in a logistic multivariate regression model for predicting the EFV decrease after SGLT2i treatment was the baseline TyG index. The TyG index represents the product of fasting glucose and triglycerides and was used for identifying insulin resistance in apparently healthy subjects [26]. Recently, the TyG index also proved to be a reliable factor in predicting the progression of coronary artery calcification [40], the incidence of prediabetes [41], and type 2 diabetes risk in adults and the elderly [42].

### Limitations

In our study, 75% of the subjects were under different dosages of statins and 19% under fenofibrate, which may influence the predictive values for the studied atherogenic biomarkers. Therefore, the power of these biomarkers for predicting the EFV decrease should also be verified in T2D patients without dyslipidemia or lipid-lowering therapy. In addition, these atherogenic biomarkers predict the EFV decrease as a dichotomous outcome. Therefore, the predictive values may change when analyzing for a specific percentage decrease in EFV. Future studies should focus on identifying pathological cut-offs of epicardial fat to define our treatment targets more efficiently. In addition, upcoming research on larger study groups and a more extended follow-up period should be performed to confirm our findings.

## 5. Conclusions

Higher initial EFV values are associated with more important reductions in EFV in patients with T2D treated for six months with dapagliflozin. TyG values have the best prediction performances for EFV reduction, having the highest sum of sensitivity and specificity at the 0.904 threshold level. AIP, CRR, VAI, conicity index, L4VF, left atrium volume, and right ventricle diameter are valid biomarkers for the EFV decrease after six months of 10 mg o.d. dapagliflozin treatment in patients with diabetes. In clinical practice, the best prediction performance for EFV reduction after dapagliflozin treatment in patients with diabetes is a TyG value > 4.93, with a sensitivity of 90.48% and specificity of 100%.

## Figures and Tables

**Table 1:** Dynamics of patients’ characteristics.

Parameter	Baseline Values	Changes after 6 Months	Δ*	*p*-Value
Weight (kg)	99.15 ± 16.07	94.13 ± 14.81	−5	<0.001 *
BMI (kg/m^2^)	34.55 ± 4.79	32.85 ± 4.76	−1.7	<0.001 *
Waist (cm)				
Men	115.03 ± 12.30	112.93 ± 11.71	−2.1	0.02 *
Women	115.05 ± 10.03	114.75 ± 11.23	-	0.92
TC (mg/dL)	192.58 ± 61.68	184.43 ± 46.45	-	0.45
TG (mg/dL)	171 (55; 887)	143 (58; 1397)	-	0.29
HDLc (mg/dL)				
Men	38 (17; 89)	42 (19; 90)	+4	0.01 *
Women	39.90 ± 12.14	45.15 ± 12.82	-	0.19
LDLc (mg/dL)	107 (50; 262)	96 (33; 202)	-	0.18
Uric acid (mg/dL)	5.16 ± 1.28	4.72 ± 1.11	-	0.07
Glycemia (mg/dL)	197 (93; 438)	155 (74; 294)	−42	<0.001 *
HbA1c (%)	8.66 ± 1.18	7.86 ± 1.26	−0.8	0.001 *
EFV (cm^3^)	37.80 ± 17.22	20.73 ± 7.09	−17.1	<0.001 *
L4VFV (cm^3^)	39.18 ± 29.17	43.27 ± 18.43	-	0.39
AIP	0.28 ± 0.33	0.20 ± 0.34	−0.08	0.04 *
CRR	4.77 (2.27; 13.08)	3.89 (2.03; 11.47)	−0.9	0.04 *
AC	3.77 (1.27; 12.08)	2.89 (1.03; 10.47)	−0.9	0.04 *
TyG index	5.24 ± 0.39	5.03 ± 0.37	−0.2	0.01 *
VAI				
Women	3.32 (1.27; 14.69)	3.15 (1.14; 29.34)	-	0.44
Men	2.81 (0.96; 22.58)	2.21 (0.47; 43.90)	-	0.20
Conicity index	1.38 ± 0.08	1.40 ± 0.07	+0.1	0.03 *
CAP (Db/m)	361 (240; 400)	310 (190; 400)	−51	<0. 001 *
Fibroscan (kPa)	6.95 (3.50; 43.80)	6.40 (3.30; 15.30)	-	0.09

* The differences are statistically significant. Continuous nonparametric numerical variables with distribution expressed as median and [interquartile range]. The results of the normal distributed variables are expressed as mean ± standard deviation. Δ* = significant differences between baseline and 6-month follow-up; BMI = body mass index; Waist = abdominal circumference; TC = total cholesterol; TG = triglyceride; HDLc = high-density lipid cholesterol; LDLc = low-density lipid cholesterol; HbA1c = glycated hemoglobin; EFV = epicardial fat volume; L4VFV = visceral fat volume at the fourth vertebrae; AIP = atherogenic index plasma; CRR = cardiac risk ratio; AC = atherogenic coefficient; TyG = product of triglycerides and glucose; VAI = visceral adiposity index; CAP = controlled attenuation parameter; Fibroscan = liver stiffness score.

**Table 2:** Correlations between atherogenic and adiposity indices and patients baseline characteristics.

Variable	Correlation Coefficient, Significance Level	AIP	CRR	TyG	VAI Women	VAI Men
Weight (kg)	*r*	−0.03	−0.01	−0.03	0.04	−0.10
	*p*	0.80	0.9	0.79	0.85	0.56
Waist (cm)	*r*	−0.08	0.75	−0.07	0.09	−0.02
	*p*	0.55	<0.001 *	0.62	0.68	0.88
BMI (kg/m^2^)	*r*	−0.12	−0.04	−0.11	0.02	−0.01
	*p*	0.40	0.76	0.42	0.92	0.94
Age (years)	*r*	−0.08	−0.24	−0.01	−0.23	0.10
	*p*	0.57	0.08	0.91	0.32	0.58
Diabetes duration (years)	*r*	−0.04	−0.21	−0.06	−0.28	0.07
	*p*	0.76	0.12	0.66	0.22	0.68
TC (mg/dL)	*r*	0.35	0.66	0.49	0.28	0.28
	*p*	0.01 *	<0.001 *	<0.001 *	0.22	0.11
TG (mg/dL)	*ρ*	0.90	0.58	0.92	0.93	0.89
	*p*	<0.001 *	<0.001 *	<0.001 *	<0.001 *	<0.001 *
HDLc (mg/dL)	*ρ*	−0.63	−0.61	−0.24	−0.70	−0.54
	*p*	<0.001 *	<0.001 *	0.08	<0.001 *	0.001 *
LDLc (mg/dL)	*ρ*	0.13	0.59	0.21	0.27	−0.07
	*p*	0.35	<0.001 *	0.13	0.24	0.67
Glycemia (mg/dL)	*ρ*	0.33	0.19	0.62	0.20	0.31
	*p*	0.01 *	0.16	<0.001 *	0.40	0.08
HbA1c (%)	*r*	0.41	0.31	0.45	0.37	0.33
	*p*	0.003 *	0.02 *	<0.001 *	0.10	0.06
Uric acid (mg/dL)	*r*	−0.08	−0.25	−0.05	−0.19	−0.16
	*p*	0.56	0.09	0.71	0.45	0.38
EFV decrease (%)	*r*	0.28	0.28	0.32	0.32	0.16
	*p*	0.04 *	0.04 *	0.01 *	0.16	0.38
EFV (cm^3^)	*r*	0.08	0.11	0.10	0.14	−0.12
	*p*	0.57	0.43	0.45	0.55	0.50
L4VFV (cm^3^)	*r*	0.10	0.58	0.15	−0.05	0.19
	*p*	0.48	<0.001 *	0.28	0.82	0.29
L4VFV decrease (%)	*ρ*	−0.03	−0.04	−0.06	−0.24	0.06
	*p*	0.81	0.77	0.65	0.29	0.73
CAP (dB/m)	*ρ*	0.21	0.09	0.24	0.33	0.14
	*p*	0.13	0.53	0.09	0.16	0.46
Fibroscan (kPa)	*ρ*	0.07	0.14	0.16	0.30	−0.04
	*p*	0.62	0.34	0.29	0.21	0.81

* The correlations are statistically significant. *r* = correlation coefficient for parametric variables; *ρ* = Spearman’s rank correlation coefficient for non-parametric variables; BMI = body mass index; Waist = abdominal circumference; TC = total cholesterol; TG = triglyceride; HDLc = high-density lipid cholesterol; LDLc = low-density lipid cholesterol; HbA1c = glycated hemoglobin; EFV = epicardial fat volume; L4VFV = visceral fat volume at the fourth vertebrae; AIP = atherogenic index plasma; CRR = cardiac risk ratio; AC = atherogenic coefficient; TyG = product of triglycerides and glucose; VAI = visceral adiposity index; CAP = controlled attenuation parameter; Fibroscan = liver stiffness score.

**Table 3:** Linear regression analysis of EFV percentage decrease and studied factors after six months of dapagliflozin.

Coefficients for EFV Decrease (%).					
Baseline Independent Variables	Unstandardized Coefficients		Standardized Coefficients	t	*p*
	B	Std. Error	Beta		
Age	−0.27	0.45	−0.09	−0.61	0.54
Diabetes duration	0.08	0.88	0.01	0.09	0.93
Weight	−0.07	0.29	−0.04	−0.26	0.79
BMI	−0.29	0.97	−0.04	−0.30	0.76
Waist	−0.58	0.40	−0.20	−1.45	0.15
TC	0.10	0.08	0.19	1.35	0.18
TG	0.04	0.03	0.22	1.55	0.12
HDLc	0.41	0.38	−0.15	−1.07	0.28
LDLc	0.14	0.10	0.20	1.40	0.16
Uric acid	−5.90	4	−0.22	−1.47	0.14
CRR	4.35	2.13	0.28	2.04	0.04 *
AIP	28.54	13.78	0.29	2.07	0.04 *
Conicity index	−119.26	55.2	−0.29	−2.16	0.03 *
TyG index	27.38	11.35	0.33	2.41	0.02 *
VAI					
Women	0.89	1.95	0.11	0.46	0.65
Men	1.70	1.32	0.23	1.29	0.20
Glycemia	0.10	0.07	0.22	1.59	0.11
HbA1c	3.96	3.86	0.14	1.03	0.31
EFV	1.38	0.18	0.73	7.50	<0.001 *
L4VFV	−0.36	0.15	−0.32	−2.38	0.02 *
L4VFV decrease (%)	−0.04	0.02	−0.31	−2.31	0.02 *
Left atrium volume	−0.97	0.32	−0.40	−3.08	0.003 *
Right ventricle diameter	−48.72	22.90	−0.33	−2.13	0.04 *
CAP	0.05	0.10	0.07	0.48	0.63
Fibroscan	0.04	0.70	0.01	0.05	0.95

* The correlations are statistically significant. BMI = body mass index; Waist = abdominal circumference; TC = total cholesterol; TG = triglyceride; HDLc = high-density lipid cholesterol; LDLc = low-density lipid cholesterol; HbA1c = glycated hemoglobin; EFV = epicardial fat volume; L4VFV = visceral fat volume at the 4th vertebrae; AIP = atherogenic index plasma; CRR = cardiac risk ratio; AC = atherogenic coefficient; TyG = product of triglycerides and glucose; VAI = visceral adiposity index; CAP = controlled attenuation parameter; Fibroscan = liver stiffness score.

**Table 4:** Receiver-operating characteristics (ROC) analysis for baseline atherogenic biomarkers and the decrease in EFV (dichotomous event).

Characteristics	AIP	CRR	TyG Index	VAI
Area under ROC	0.903	0.772	0.957	0.898
*p* value	<0.001	0.004	<0.001	<0.001
Youden index J	0.732	0.595	0.904	0.712
Associated criterion	>0.02	>4.03	>4.93	>1.98
Sensitivity	85.71%	72.09%	90.48%	83.72%
Specificity	87.50%	87.50%	100%	87.50%

ROC = receiver-operating characteristics; AIP = atherogenic index plasma; CRR = cardiac risk ratio; TyG = product of triglycerides and glucose; VAI = visceral adiposity index.
